# Perioperative nursing outcomes and management strategies in cold plasma ablation for superficial corneal disorders

**DOI:** 10.3389/fsurg.2026.1665131

**Published:** 2026-06-10

**Authors:** Dan Hu, Yuan Lin, Huping Wu

**Affiliations:** 1 Xiamen Eye Center and Eye Institute of Xiamen University, School of Medicine, Xiamen, China; 2 Xiamen Clinical Research Center for Eye Diseases, Xiamen, Fujian, China; 3 Xiamen Key Laboratory of Ophthalmology, Xiamen, Fujian, China; 4 Fujian Key Laboratory of Corneal & Ocular Surface Diseases, Xiamen, Fujian, China; 5 Xiamen Key Laboratory of Corneal & Ocular Surface Diseases, Xiamen, Fujian, China; 6 Translational Medicine Institute of Xiamen Eye Center of Xiamen University, Xiamen, Fujian, China

**Keywords:** cold plasma ablation, corneal epithelium, epithelial healing, ocular surface surgery, perioperative nursing, superficial keratectomy

## Abstract

**Objective:**

To evaluate the clinical outcomes and perioperative nursing outcomes of low-temperature plasma ablation (LTPA) for the treatment of superficial corneal diseases.

**Methods:**

This retrospective study analyzed 38 patients who underwent LTPA between June 2022 and January 2024. Patients were grouped by etiology into refractive/structural (*n* = 30) and infectious (*n* = 8) categories. Clinical outcomes, epithelial healing time, complications, pain scores, and digital slit-lamp image-based healing assessments were observed and compared between the two groups. Pain intensity was longitudinally monitored using visual analog scale (VAS) scores and analyzed via two-way repeated measures ANOVA. Perioperative nursing strategies and patient-reported satisfaction were also evaluated.

**Results:**

The study population consisted of 38 patients (including 16 males and 22 females), with a mean age of 51.97 ± 2.36 years (range: 22–87 years). Patients in the infectious group exhibited significantly longer epithelial healing times compared to those in the noninfectious group (*p* = 0.040). Recurrence within 3 months was significantly more frequent in the infectious group (25.0%, *n* = 2/8 vs. 0%, *n* = 0/30; *p* = 0.005) and was associated with lower nursing satisfaction scores (*p* < 0.001). Two-way repeated measures ANOVA revealed that VAS pain scores were consistently higher in the infectious group across all time points (*F* = 20.33, *p* < 0.001), although both groups demonstrated a significant downward trend over time (*F* = 241.10, *p* < 0.001). Quantitative analysis based on digital slit-lamp imaging facilitated the clinical observation delayed epithelial healing in 5 patients (13.2%), thereby facilitating early clinical intervention. No severe postoperative complications were reported.

**Conclusion:**

LTPA appears to be a feasible and well-tolerated approach for treating superficial corneal disorders, associated with preliminary outcomes being observed across both infectious and noninfectious etiologies. These findings suggest that perioperative nursing protocols and digital image-based monitoring may support objective healing tracking and enhance patient care, although these observational results require prospective validation.

## Introduction

Superficial keratectomy (SK) is a major surgical intervention for patients with superficial corneal diseases that are unresponsive to conventional medical therapy (1). The procedure involves the mechanical removal or excimer laser ablation of the superficial corneal layers, including the epithelium, Bowman's membrane, and the anterior stroma (on occasion) (2–4). To improve surgical outcomes, some surgeons have incorporated adjunctive measures such as 0.02% mitomycin-C application or amniotic membrane transplantation during the procedure (5, 6). SK remains effective at managing a range of conditions, including tissue biopsy, corneal degeneration, corneal dystrophy, scarring, recurrent epithelial erosions, and residual superficial foreign bodies (7). However, this study demonstrated several limitations. Common postoperative complications include corneal haze, infection, and delayed epithelial healing. Moreover, SK is contraindicated in patients with active keratitis or in those with inadequate corneal thickness (8).

To address these limitations and expand treatment indications, low-temperature plasma ablation (LTPA) has recently been introduced as an alternative surgical technique for the ocular surface. LTPA replaces traditional mechanical instruments (such as surgical blades or epithelial scrapers) with the use of a plasma-mediated approach for more precise and complete removal of pathological tissue. By applying radiofrequency energy to an electrolyte solution around the electrode, the low-temperature plasma system generates a thin plasma layer that is approximately 50 μm thick. This plasma layer breaks molecular bonds within the target tissue at low temperatures, thus allowing for efficient tissue ablation, disintegration, and coagulation while minimizing collateral damage (9, 10). Due to the fact that no direct thermal cutting occurs and current does not pass through the tissue, the surrounding structures experience minimal heating and damage, thus demonstrating this technique as being particularly advantageous for delicate ocular tissues (11).

By utilizing low-temperature radiofrequency plasma, this technique enables ablation, cutting, and hemostasis at significantly lower temperatures, thereby reducing the risk of iatrogenic injury (12). In ophthalmology, the applications of LTPA have expanded, with reports supporting its use in surgeries for pterygium, recurrent corneal epithelial erosion, infectious corneal ulcers, and conjunctival cysts. Its efficacy and safety have also been demonstrated in both noninfectious and fungal corneal diseases (13–15). The present study aimed to summarize our perioperative nursing experiences and clinical outcomes in patients undergoing LTPA for superficial corneal lesions.

Although the surgical efficacy of LTPA has been explored, its integration with objective healing assessments and specialized care remains undocumented. The novelty of this study involves the proposal of a comprehensive, multidisciplinary clinical pathway combining the minimally invasive nature of LTPA with innovative R-based digital image quantification for objective healing tracking (supported by tailored perioperative nursing). Therefore, the present study aimed to not only evaluate the clinical outcomes of LTPA in patients with infectious and noninfectious superficial corneal lesions but also establish a data-driven, perioperative management model.

## Methods

### Patients

This retrospective study was approved by the Ethics Committee of Xiamen Eye Center affiliated with Xiamen University. We reviewed the medical records of 38 patients who underwent LTPA for superficial corneal lesions between June 2022 and January 2024. The cohort included 16 males and 22 females, with a mean age of 51.97 ± 2.36 years (range: 22–87 years).

### Inclusion and exclusion criteria

The following inclusion criteria were utilized: (1) superficial corneal lesions limited to the anterior one-third of the stroma (depth <150 μm) that were objectively confirmed via anterior segment optical coherence tomography (AS-OCT); (2) failure of conventional medical therapy for at least 2 weeks; and (3) a documented follow-up of at least 3 months. In the infectious group, acute suppuration had to be controlled prior to surgery.

The exclusion criteria included patients with uncontrolled deep stromal or endothelial involvement, endothelial decompensation, severe corneal thinning (<250 μm), uncontrolled uveitis, or systemic autoimmune disorders affecting wound healing.

### Clinical data collection

Demographic and clinical data, including sex, age, underlying etiology of corneal disease, percentage of epithelial defects, and postoperative pain score, were collected from all the patients by using a visual analog scale (VAS).

### Image-based assessment of epithelial healing

Postoperative corneal epithelial healing was quantitatively evaluated by using digital slit-lamp images captured under cobalt blue illumination after fluorescein staining. The images were processed by using R (version 4.3.2) was employed as a standardized framework for the clinical tracking of the healing process. Postoperative epithelial defects were quantified by using a standardized digital workflow. Briefly, fluorescein-stained images were processed via an automated R-based algorithm to isolate the green fluorescence channel and delineate the defect area. To address the absence of formal multi-center validation, all image analyses were initially performed by a single operator (D.H.) and subsequently double-checked by a second investigator (Y.L.). Internal consistency was maintained by visually confirming the R algorithm's automated defect boundaries against the original raw images for a randomly selected 20% subset of patients to ensure clinical utility in our cohort. However, formal validation metrics such as interobserver agreement or test-retest reproducibility were not quantitatively assessed in this study.

### Treatment strategy

All surgeries were performed under peribulbar anesthesia by using a low-temperature plasma system (PLA-700, Meichuang Medical, Chengdu, China) with an MC-409 probe. Lesion depth and extent were determined by using slit-lamp evaluation, anterior segment OCT, and *in vivo* confocal microscopy. Postoperative care included topical antibiotics, steroids, and epithelial growth-promoting agents. Daily slit-lamp evaluations were used to assess healing, inflammation, and complications. Nursing measures included preoperative education, perioperative asepsis, and tailored discharge instructions. Patients were followed for at least 3 months after the operation.

### Nursing strategy

All patients included in this retrospective analysis received structured and individualized perioperative nursing management as part of routine care during LTPA for superficial corneal lesions. The nursing strategy was designed to optimize surgical outcomes, reduce postoperative complications, and enhance patient satisfaction through a standardized but personalized approach.

Preoperative care began with comprehensive patient assessment by a multidisciplinary team comprising ophthalmologists, anesthesiologists, and primary care nurses. The evaluation included corneal lesion characteristics, systemic conditions (such as diabetes and autoimmune diseases), and psychosocial factors. Patients and their families received individualized education using verbal explanations and visual aids to ensure a full understanding of the surgical procedure, potential risks, and expected recovery. Psychological counseling was provided as needed to reduce anxiety and improve compliance. Intraoperatively, nurses ensured strict aseptic technique, monitored vital signs, and provided real-time emotional support. Special attention was given to maintaining the consistent operation of the LTPA device, including the monitoring of plasma output and irrigation flow. Postoperatively, nurses evaluated the healing process on a daily basis using slit-lamp photography. Digital images were analyzed and quantified to assess the epithelial defect area and detect delayed healing. These measurements were cross-checked with physicians and recorded in nursing notes to guide medication adjustments and follow-up planning.

Patients were counseled on eye hygiene, avoidance of eye rubbing, and proper use of medications. Nutritional guidance emphasized the importance of high-protein, vitamin-rich soft foods to support tissue repair. Pain, photophobia, and discomfort were managed according to standardized protocols. Discharge planning involved tailored education on medication schedules, eye care precautions, and activity restrictions. Follow-up visits were scheduled at 2 weeks, 1 month, and 3 months. High-risk patients (such as those with infectious ulcers or systemic comorbidities) were monitored via telephone to ensure the early detection of complications and maintain adherence.

### Statistical analysis

All of the statistical analyses were performed by using GraphPad Prism (version 8.4.3; GraphPad Software, San Diego, CA, USA). Continuous variables are expressed as the mean ± standard deviation (SD) and were compared by using unpaired *t*-tests when the data were normally distributed. Categorical variables (including the sex ratio, recurrence rate, delayed epithelial healing, and nursing satisfaction) were compared by using the chi-square test or Fisher's exact test, as appropriate. For comparisons of VAS scores between the infectious and noninfectious groups at multiple time points (including preoperative, POD1, POD3, and POD7 time points), a two-way repeated measures analysis of variance (ANOVA) was performed, followed by Bonferroni's *post hoc* test for multiple comparisons. A *p* value < 0.05 was considered to indicate statistical significance.

Image-based epithelial area quantification derived from digital slit-lamp photography was processed by using standard R-based image analysis protocols. By focusing on the green channel (which best captures fluorescein emission under cobalt blue light) and employing Otsu's thresholding, we ensured consistent delineation of the defect margins across the patients.

## Results

A total of 38 patients (38 eyes) were included in the analysis and categorized into the noninfectious group (*n* = 30) and the infectious group (*n* =8) (Table 1). No significant differences were observed between the two groups in terms of age (50.87 ± 14.29 vs. 56.13 ± 15.80 years, *p* = 0.923) or sex distribution (*p* = 0.194). However, patients in the infectious group exhibited significantly longer epithelial healing times compared to the refractive group (13.50 ± 4.53 vs. 5.87 ± 2.44 days, *p* = 0.040). Additionally, recurrence within 3 months was significantly more frequent in the infectious group (25.0%, *n* = 2/8 vs. 0%, *n* = 0/30; *p* = 0.005). Nursing satisfaction was also lower in the infectious group (75.50% vs. 96.66%, *p* < 0.001) (Table 2).

**Table 1:** Etiological classification of patients undergoing low-temperature plasma ablation (LTPA) for superficial corneal lesions.

Group	Number of patients (n)	Subtype	*n* (%)
Noninfectious group	30	Recurrent corneal erosion	13 (43.3%)
		Corneal epithelial hyperplasia	7 (23.3%)
		Corneal degeneration	7 (23.3%)
		Superficial stromal scarring	2 (6.7%)
		Epithelial basement membrane dystrophy	1 (3.3%)
Infectious group	8	Infectious corneal ulcers	8 (100%)

**Table 2:** Demographic and clinical characteristics of patients undergoing Low-temperature plasma ablation (*n* = 38).

Variable	Noninfectious group (*n* = 30)	Infectious group (*n* = 8)	*p* value
Age (years, mean ± SD)	50.87 ± 14.29	56.13 ± 15.80	0.923
Sex (male:female)	11:19	5:3	0.194
Epithelial healing time (days)	5.87 ± 2.44	13.50 ± 4.53	0.040*
Recurrence within 3 months	0 (0%)	2 (25.0%)	0.005**
Nursing satisfaction (%)	96.66% (29/30)	75.50% (6/8)	<0.001***

* *p* < 0.05.

** *p* < *0.01*.

*** *p* < 0.001.

**Figure 1:**
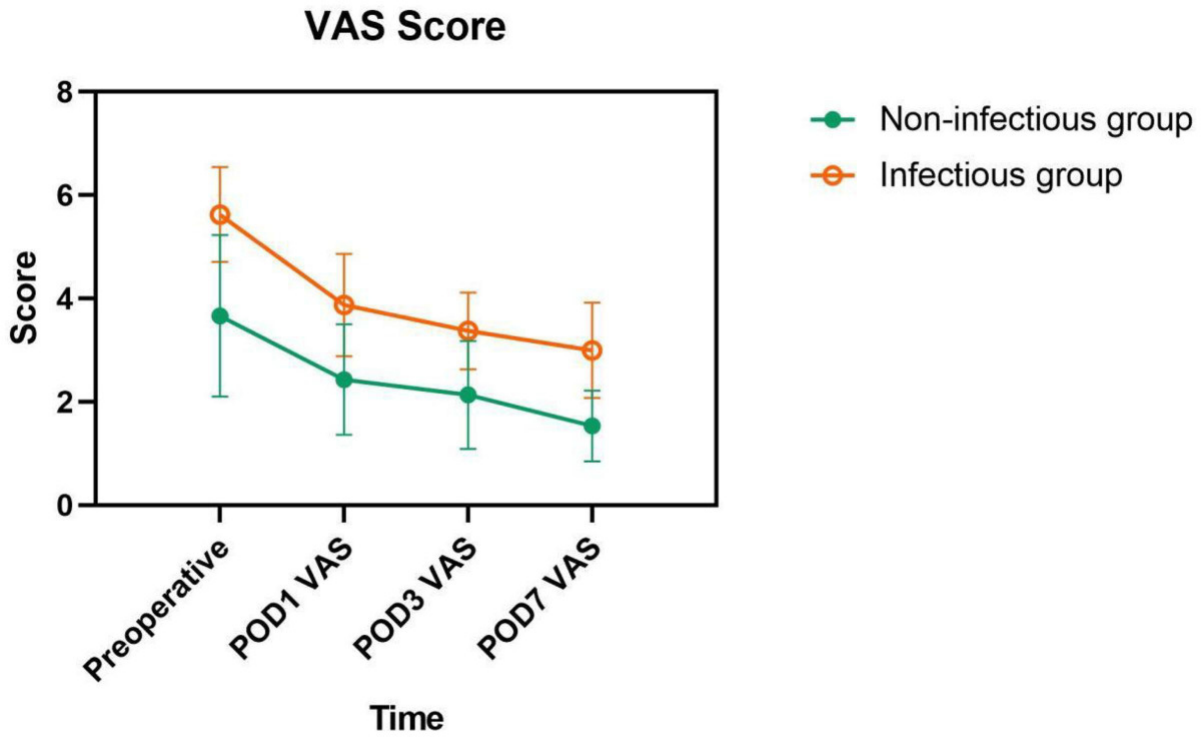
Comparison of postoperative pain scores (VAS) between the infectious and noninfectious groups. VAS scores were recorded preoperatively and on postoperative days (POD) 1, 3, and 7 for the infectious and noninfectious groups. Analysis using a two-way repeated measures ANOVA demonstrated a significant main effect for group (*F* = 20.33, *p* < 0.001) and time (*F* = 241.10, *p* < 0.001), whereas the group-by-time interaction was not significant (*F* = 1.95, *p* = 0.120). *post hoc* comparisons with Bonferroni correction confirmed that the infectious group consistently exhibited significantly higher pain scores at all time points (preoperatively, *p* < 0.001; POD1, *p* = 0.001; POD3, *p* = 0.003; and POD7, *p* < 0.001). Data are presented as the mean ± standard deviation (SD).

Postoperative discomfort (as measured via the VAS score) was also lower in the noninfectious group than in the infectious group (Figure 1). To account for multiple temporal comparisons of VAS scores (ranging from preoperative to POD7 time points), a Bonferroni correction was applied, with the significance threshold set at *p* < 0.05. Repeated measures ANOVA revealed a significant main effect for group (*F* = 20.33, *p* < 0.001) and time (*F* = 241.10, *p* < 0.001). The interaction between time and group was not significant (*F* = 1.95, *p* = 0.120), thus indicating a similar pattern of pain reduction in both groups. *post hoc* tests with Bonferroni correction confirmed that patients in the infectious group reported significantly higher VAS scores at all of the observed time points (all *p* < 0.01).

During the 3-month follow-up period, five patients (13.2%) exhibited delayed epithelial healing (including four patients in the infectious group and one patient in the noninfectious group). Additionally, two patients in the infectious group (25%) developed recurrent epithelial defects, whereas no recurrences were observed in the noninfectious group. Among the infectious group, two patients were diagnosed with fungal keratitis. Despite undergoing low-temperature plasma ablation and receiving standard postoperative treatment, both patients demonstrated progressive clinical deterioration. As a result, one patient required therapeutic penetrating keratoplasty, and the other patient underwent conjunctival flap coverage surgery to maintain globe integrity.

**Figure 2:**
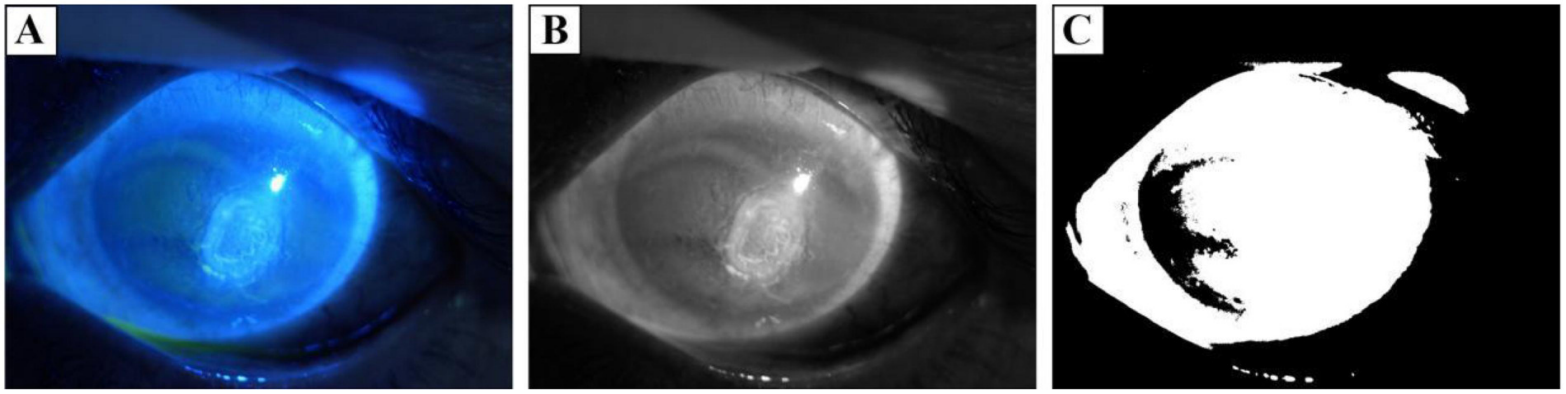
Digital slit-lamp image-based evaluation of corneal epithelial defects. Representative fluorescein-stained slit-lamp image processed through a customized R-based (version 4.3.2) digital workflow for objective healing assessment. **(A)** Original image captured under cobalt blue illumination demonstrating fluorescein uptake at the corneal epithelial defect site. **(B)** Extraction of the green color channel to optimize fluorescence contrast against the background. A binary threshold was then applied by using Otsu's method to distinguish the defect area (white pixels) from the intact epithelium (black pixels). **(C)** Region-specific quantitative analysis. Artifacts such as eyelid margins and light reflections were excluded, with only intracorneal fluorescein signals quantified to ensure accuracy. In this example, the identified defect area accounted for 5,716 pixels (21.27%) of the total corneal surface.

**Figure 3:**
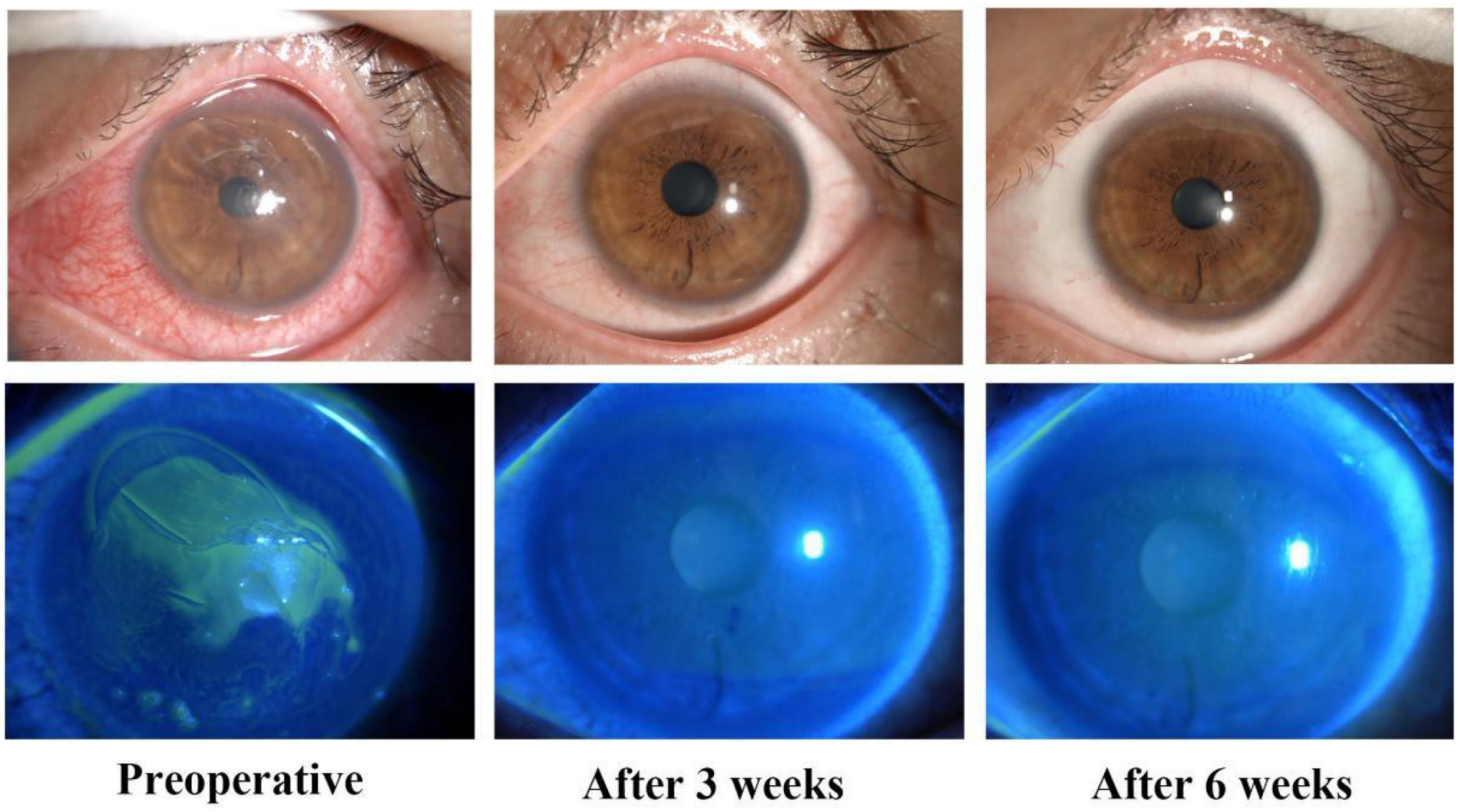
Follow-up of patients discharged after receiving LTPA. The corneal epithelium of patients with continuous follow-up demonstrated good repair; moreover, the inflammatory response continued to decrease, and there was no recurrence.

Digital slit-lamp imaging was successfully incorporated in all of the patients to postoperatively assess the epithelial defect area (Figure 2). Image quantification enabled the earlier detection of nonhealing zones in 4 of the 5 delayed-healing cases. This approach enhanced the precision of nursing documentation and facilitated timely adjustments of topical medications. This image-based analysis method was applied to all of the patients during early postoperative follow-up (days 1–3) to assess the extent and progression of epithelial healing. In 5 patients (13.2%) with delayed healing, image analysis enabled the early recognition of stagnation in epithelial defect resolution. This facilitated timely adjustments of medications. Finally, patients who underwent LTPA achieved significant pain relief, and no recurrence was observed at the subsequent follow-up (Figure 3).

## Discussion

This observational study highlights the clinical potential of LTPA as a precise and minimally invasive approach for managing various superficial corneal disorders. Our findings suggest that LTPA provides a controlled, tissue-saving alternative to conventional SK, particularly in challenging cases such as those involving thin corneas or recalcitrant ulcers for which traditional mechanical debridement may exhibit greater risks. This technique demonstrates favorable outcomes, including effective lesion removal, rapid epithelial recovery, and a low incidence of complications (16). In particular, traditional SK poses greater risks, such as thin corneas or nonhealing ulcers (17). LTPA provides a controlled, tissue-saving option. Low-temperature plasma ablation + drug therapy can effectively control disease progression and significantly shorten the duration of ulcer healing (18). Notably, the primary intent of stratifying patients by etiology (infectious vs. noninfectious patients) was to delineate their distinct postoperative healing profiles (rather than to establish comparative efficacy). Our results demonstrated that infectious keratitis was associated with significantly prolonged healing times and elevated VAS pain scores following LTPA. This outcome underscores the necessity of risk-stratified nursing interventions and intensive follow-up protocols tailored to the underlying pathology. Ultimately, the outcomes observed in this study reflect the synergy of the surgical technique and a specialized perioperative nursing pathway, thus suggesting that the integration of both components is essential for optimizing the recovery of the ocular surface.

Evidence-based nursing for postoperative patients can significantly improve the recovery of patients' postoperative vision and reduce the incidence and recurrence rate of postoperative complications. Moreover, it is also helpful for improving patients' adverse psychological states and quality of life after surgery (19). The integration of digital fluorescein image analysis into the nursing workflow provided a quantitative, reproducible, and objective method of assessing epithelial recovery. This approach minimized potential subjectivity, improved documentation accuracy, and enhanced interdisciplinary communication. Nursing staff were successfully trained to perform image-based assessments, with the results demonstrating internal verification with evaluations conducted by ophthalmologists.

Notably, early signs of delayed epithelial healing were detected in 4 out of 5 patients through the use of a digital monitoring system, thus highlighting its clinical utility during the early postoperative period. These image-based tools were particularly effective at identifying subtle nonhealing zones, thereby enabling timely medication adjustments and supporting personalized postoperative care. Furthermore, no serious complications (such as corneal perforation, endothelial decompensation, or secondary infection) were observed. Together, these findings emphasize the synergistic value of integrating LTPA with structured, data-driven nursing care to improve clinical outcomes, reduce complication risks, and increase patient satisfaction.

A key driver of the favorable outcomes observed in this study was the implementation of a structured, individualized perioperative nursing protocol. This model encompasses comprehensive preoperative assessment, standardized intraoperative collaboration, and personalized postoperative follow-up (20). Prior to surgery, nurses conducted detailed evaluations of lesion type, depth, and systemic comorbidities, which informed the design of risk-stratified, patient-specific care plans (21). These assessments not only optimize surgical preparedness but also allow nurses to anticipate complications and personalize postoperative regimens (22). Patient education was a cornerstone of the preoperative phase and aimed to reduce anxiety, improve the understanding of the surgical process, and enhance medication adherence. During surgery, nursing care focused on maintaining a sterile field, verifying the functionality of the LTPA equipment, and supporting patient comfort and psychological stability. The coordinated intraoperative workflow between the nursing staff and surgeons ensured procedural precision and minimized complications.

Postoperatively, nurses implemented phase-specific interventions tailored to individual healing trajectories. Key priorities included the daily monitoring of corneal epithelial recovery, control of inflammation, and early detection of complications (23). Importantly, this study integrated digital slit-lamp photography and image-based analysis into the nursing workflow, thereby enabling the objective and quantifiable assessment of epithelial defects. This approach facilitated timely medication adjustments, reduced interobserver potential subjectivity, and strengthened interdisciplinary communication. Moreover, it represents a scalable model of precision nursing aligned with smart health care paradigms.

Our results also revealed that disease subtype influences recovery. Patients with infectious keratitis (particularly fungal ulcers) exhibited significantly longer healing times than those with noninfectious etiologies such as recurrent epithelial erosion or corneal degeneration. This finding emphasizes the need for tailored nursing interventions and more intensive follow-up protocols for high-risk patients (24). Throughout all phases of care, psychological support was continuously integrated. From preoperative counseling and intraoperative communication to discharge planning, nurses employed empathy-driven strategies to alleviate emotional distress and improve compliance (25). Postdischarge instructions were reinforced by using illustrated handouts, video tutorials, and scheduled follow-ups (both in person and via digital platforms) for patients requiring closer monitoring.

Several limitations of this study should be acknowledged. First, its retrospective and observational design introduces inherent selection bias and precludes the establishment of a definitive causal relationship between the interventions and clinical outcomes. Second, the total sample size was relatively small (*n* = 38), and the infectious subgroup (*n* = 8) was particularly limited, which reduces the statistical power and generalizability of the findings across broader clinical populations. Third, the lack of a comparator group represents a core limitation that significantly affects the interpretability of the results. Without a randomized control, these data alone indicate that LTPA should be considered as a feasible and well-tolerated alternative rather than a demonstrably superior technique based on these data alone. Additionally, the follow-up duration of three months was relatively short, and longer-term studies are necessary to fully assess recurrence rates and the long-term stability of the ocular surface. With respect to digital image analysis, although it provides an objective workflow for tracking epithelial healing, this workflow has not been formally validated for interobserver or multicenter reproducibility. We acknowledge that while our R-based digital analysis offers a more structured approach than visual estimation, it lacks formal validation through metrics such as Cohen's kappa or intraclass correlation coefficients. Future studies with multi-center data are required to establish its robust precision.

Finally, although the results are promising, future prospective, randomized controlled trials are warranted to validate these preliminary findings and to standardize LTPA protocols, including optimal energy settings and postoperative medication strategies. Despite these constraints, the potential for machine learning-based analysis of slit-lamp images represents an exciting frontier in automating and personalizing postoperative care in ophthalmic surgery.

## Conclusion

Low-temperature plasma ablation is a feasible option for treating superficial corneal diseases with favorable preliminary outcomes that require validation in prospective trials. When combined with standardized perioperative nursing care (including thorough assessment, strict infection control, and structured follow-up), it promotes faster recovery, fewer complications, and greater patient satisfaction. This integrative approach may serve as a valuable model for enhancing outcomes in ocular surface surgery.

## Data Availability

The original contributions presented in the study are included in the article/Supplementary Material, further inquiries can be directed to the corresponding author/s.
