# Supplementary material for: Perioperative nursing outcomes and management strategies in cold plasma ablation for superficial corneal disorders
Source: Front Surg. 2026 Jun 10;13:1665131. doi: 10.3389/fsurg.2026.1665131 (PMC13291153; doi:10.3389/fsurg.2026.1665131)
Supplement: Supplementary file 1 [file Supplementaryfile1.docx]

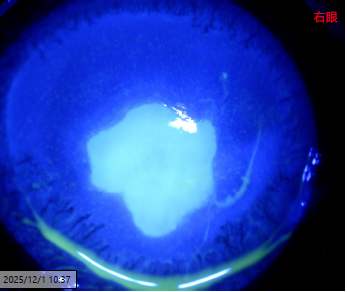

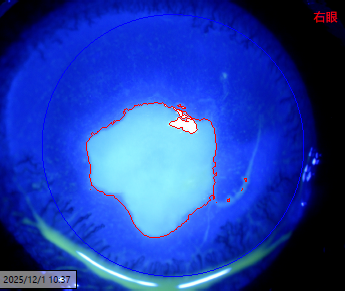


Supplementary Method 1: R-Based Digital Image Analysis Workflow for Corneal Epithelial Defects

In this study, the quantitative assessment of the corneal epithelial defect area was performed by using an automated image processing workflow based on R (version 4.3.2) and the EBImage package. Compared with traditional visual estimation, this digital workflow eliminates interobserver variability and provides standardized framework for the clinical tracking of the healing process. The specific image analysis steps are detailed below.

1. Image Acquisition and Preprocessing

All of the postoperative corneal images were captured in a standard darkroom environment by using a digital slit-lamp biomicroscope (with a cobalt blue filter) in a uniform manner after the instillation of sodium fluorescein. The acquired standard RGB images were first cropped to remove interference from extraocular tissues such as eyelid margins, eyelashes, and scleral reflections, thereby retaining only the effective total corneal area as the baseline for subsequent analysis.

2. Green Channel Extraction

Under cobalt blue light excitation (wavelength of ~490 nm), sodium fluorescein emits a yellow-green fluorescence (peak emission wavelength of ~520 nm). In the RGB (red, green, and blue) color model of the digital images, the signal from the fluorescein-stained area is predominantly concentrated in the green channel. Therefore, the first step in image processing uses the EBImage package to separate the color channels of the original image and extract the grayscale matrix corresponding specifically to the green channel. This step not only maximizes the visibility of the lesion but also effectively filters out background noise caused by conjunctival hyperemia (red signal) and corneal reflections (blue/white signals).

3. Image Segmentation via Otsu's Method

To precisely separate the stained “epithelial defect area” (foreground) from the unstained “intact epithelial area” (background), this study employed Otsu’s thresholding method. This algorithm is a classic global adaptive thresholding technique that exhaustively searches the image’s grayscale histogram to calculate the interclass variance and identify the optimal grayscale threshold that maximizes the difference between the foreground and background.

Based on the calculated Otsu threshold, the extracted green channel image is converted into a binary mask. In this mask, pixels with values above the threshold are assigned a value of 1 (white, which represents the epithelial defect area), and pixels below the threshold are assigned a value of 0 (black, which represents the intact epithelial area).

4. Pixel-Based Area Quantification

After the binary mask is obtained, the system automatically counts the total number of pixels marked as white (defect pixels) and calculates their proportion relative to the total number of pixels in the entire effective corneal area. The final extent of epithelial loss is expressed as the percentage (%) of foreground pixels out of the total pixels.

5. Quality Control and Clinical Collaboration

To ensure the clinical validity of the measurement results, the specialized nursing staff who participated in this study received standardized training on this R-based analysis workflow. In practical clinical applications, nurses were responsible for the initial importation and automated analysis of the images. The attending ophthalmologist subsequently conducted a double-blind cross-validation of the boundaries in the generated binary images against the original clinical images. This dual-safeguard mechanism of “algorithmic quantification + collaborative medical-nursing verification” ensured both data accuracy and consistency in clinical decision-making.
